# Supplementary material for: Specific Induction of Double Negative B Cells During Protective and Pathogenic Immune Responses
Source: Front Immunol. 2020 Dec 18;11:606338. doi: 10.3389/fimmu.2020.606338 (PMC7775384; doi:10.3389/fimmu.2020.606338)
Supplement: Supplementary file 1 [file DataSheet_1.pdf]

Supplementary Materials:

Supplementary Figure S1: Gating Strategy including FMO controls

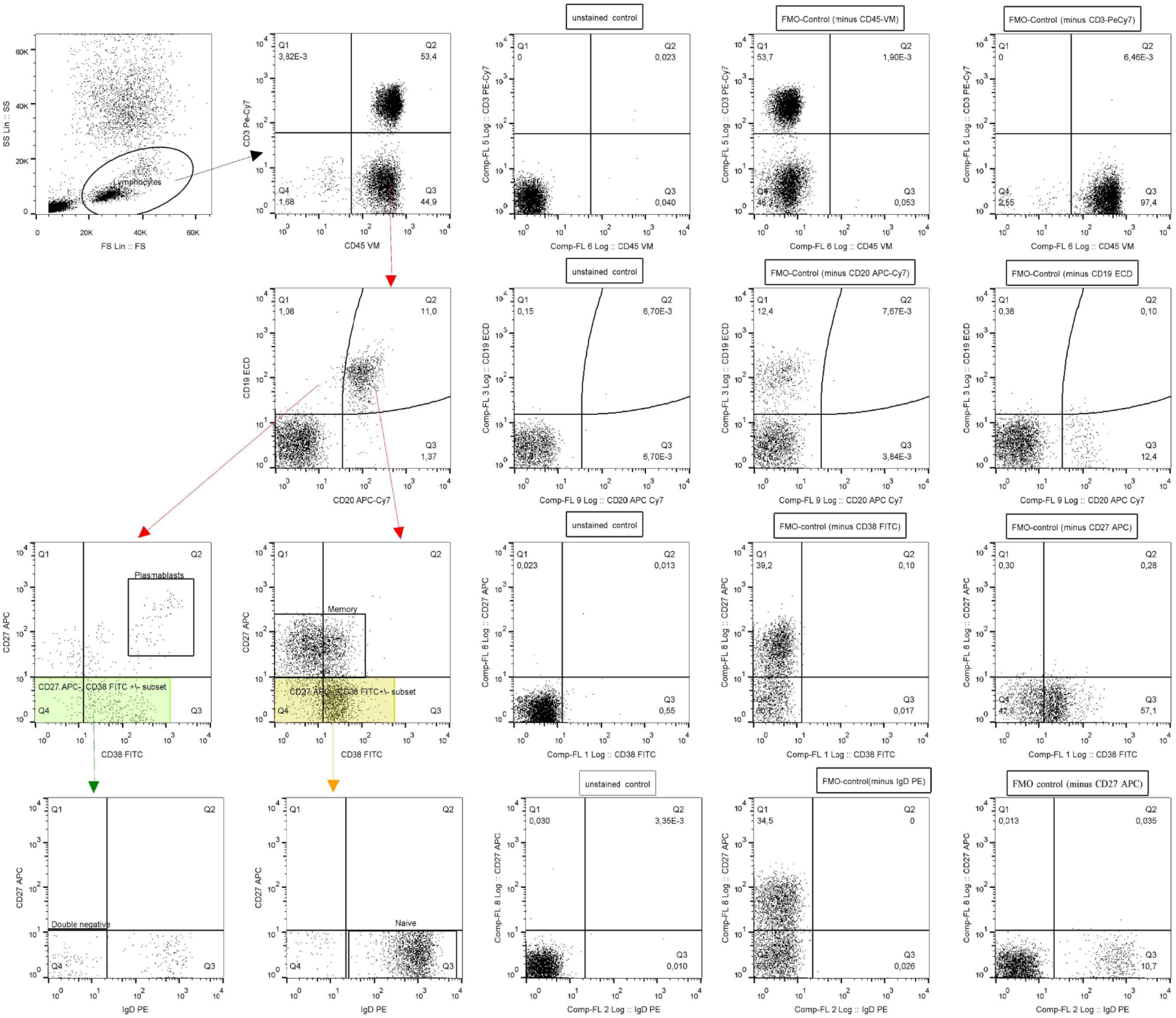

Fig. S1: Gating strategy (columns 1 and 2) for differentiation of B cell subsets. Column 3 displays unstaiend control, column 4 and 5 FMO (fluorescence minus one) control, each missing the corresponding fluorochrome for x-axis (column 4) or y-achsis (column 5). B cell populations are defined as followed: naïve B cells CD19^{+}CD20^{+}CD27^{-}CD38^{+}IgD^{+}, memory B cells CD19^{+}CD20^{+}CD27^{+}CD38^{+}, double negative B cells (DN B cells) CD19^{+}CD20l^{ow}CD27^{-}IgD^{-}, plasmablasts CD19^{+}CD20^{low}CD27^{+}CD38^{high}IgD^{-}.

Supplementary Figure S2: B cell subsets in MS cohort and differentiation of plasmablasts

A)

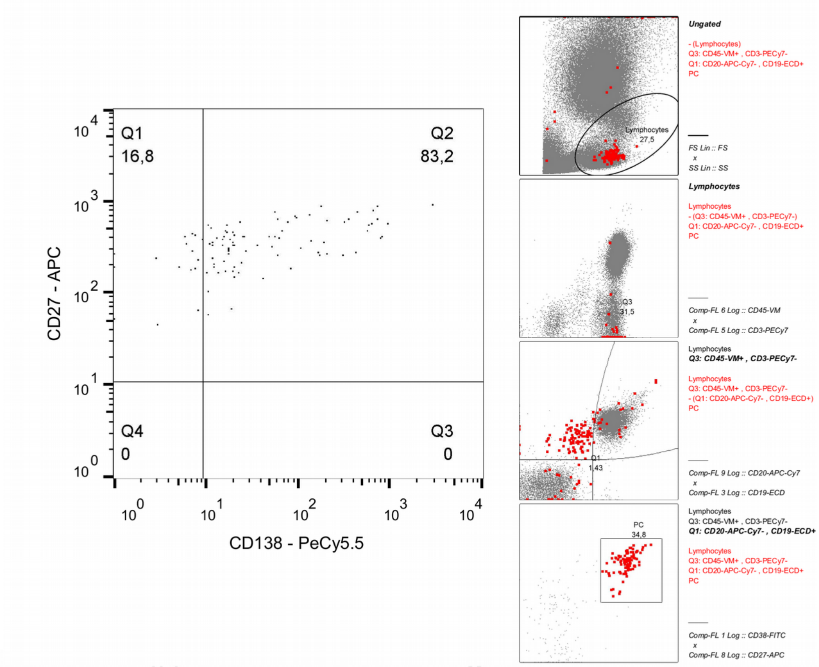

B)

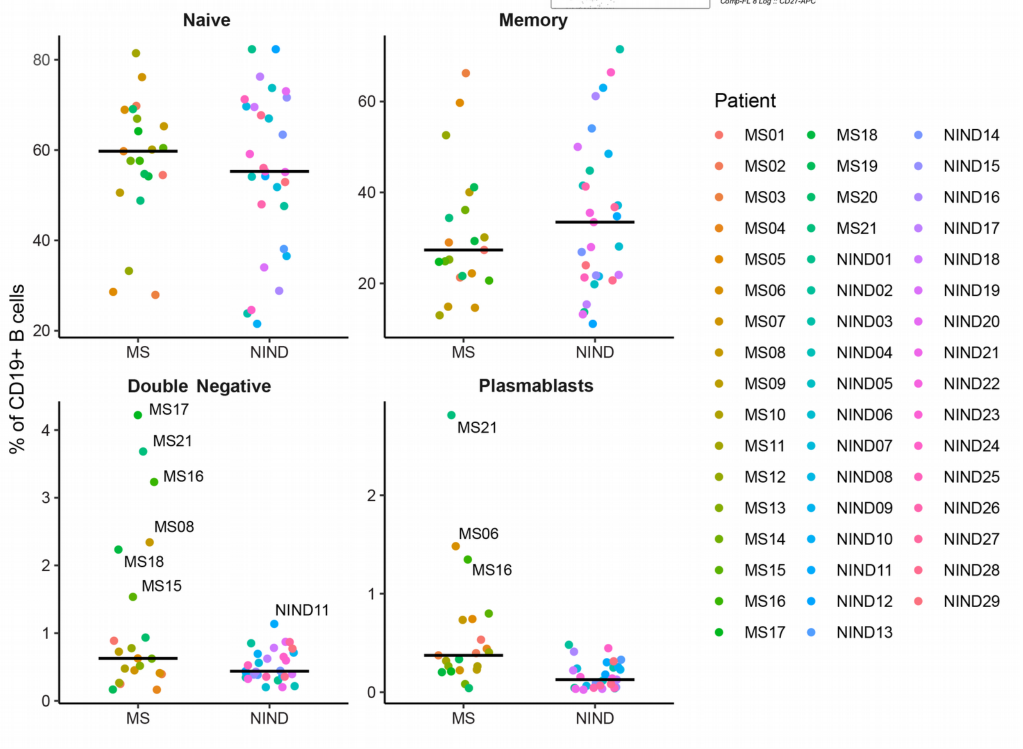

Fig. S2: A) Exemplary staining (untreated healthy control) with CD138-PeCy5.5 confirms that >80% of CD45+CD3-CD19+CD20^{low}CD27+CD38^{high} also express CD138+. Ancestry on the right column with backgating of PB displays features of PB in FSC vs SSC, CD3 vs CD45 and CD19 vs CD20.B) Detailed display of MS cohort (color coded) for B cell subsets. Clinical characteristics of each patient is displayed in Supplementary Table S1

Supplementary Figure S3

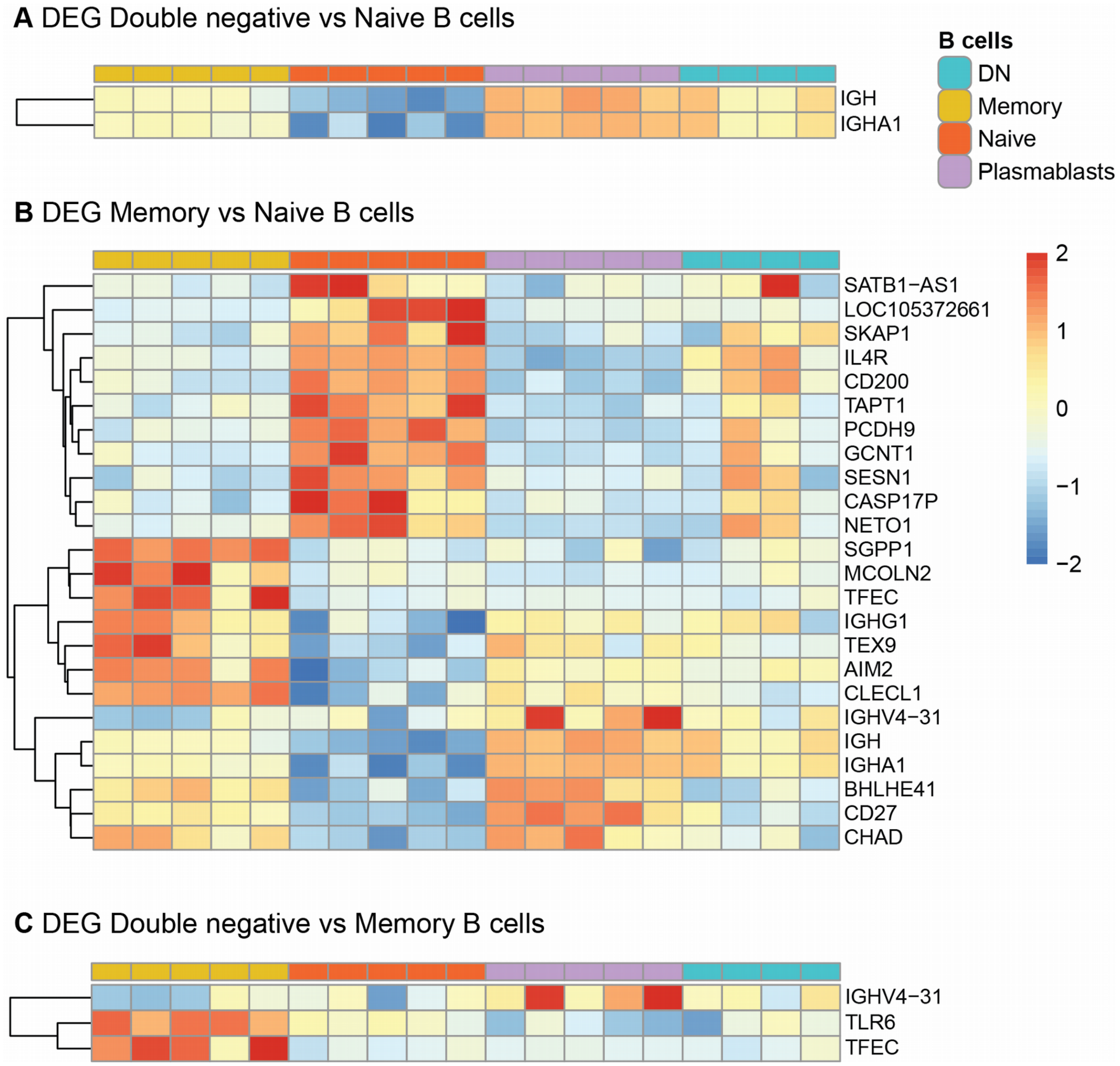

Fig. S3: Normalized gene expression for DEG in 3 different contrasts: (A) DN B-cells vs Naïve B-cells, (B) Memory vs Naïve B-cells, and (C) DN vs Memory B-cells. The hierarchical clustering dendrogram of the genes according to their expression patterns in the different samples is shown at the left. The color scale shows the normalized gene expression values scaled between -2 and 2 for each gene (row).

Supplementary Figure S4

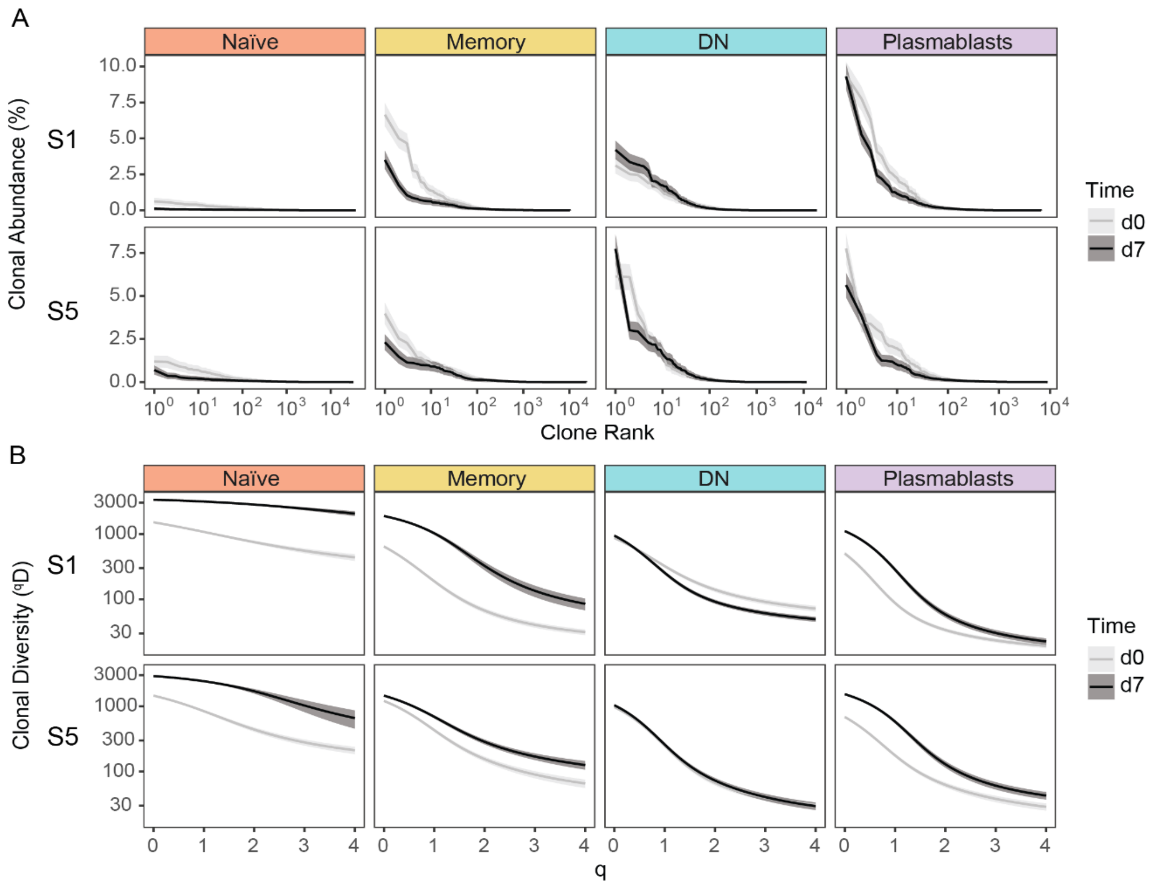

Fig. S4: Characteristics of the clonal repertoire of subjects 1 (S1) and 5 (S5), at day 0 before and day-7 after TBE vaccination. (A) Clonal abundance of the naïve, memory, DN and plasmablast repertoires. Clones were ranked according to decreasing sizes from left to right, and their relative abundance as percentage of the V(D)J sequence repertoire is represented. (B) Clonal diversity of the repertoire represented with Hill numbers (^{q}D) at different orders (q). For both clonal abundance and diversity calculations, a sampling of the unique sequence repertoire was performed, selecting N = 3906 unique sequences, with n = 1000 bootstrap iterations. This ensures the same number of sequences in each of the time points, and allows for correction of library sequencing depth correction. The gray area on both curves indicates the variability among the bootstrap samples.

Supplementary Figure S5

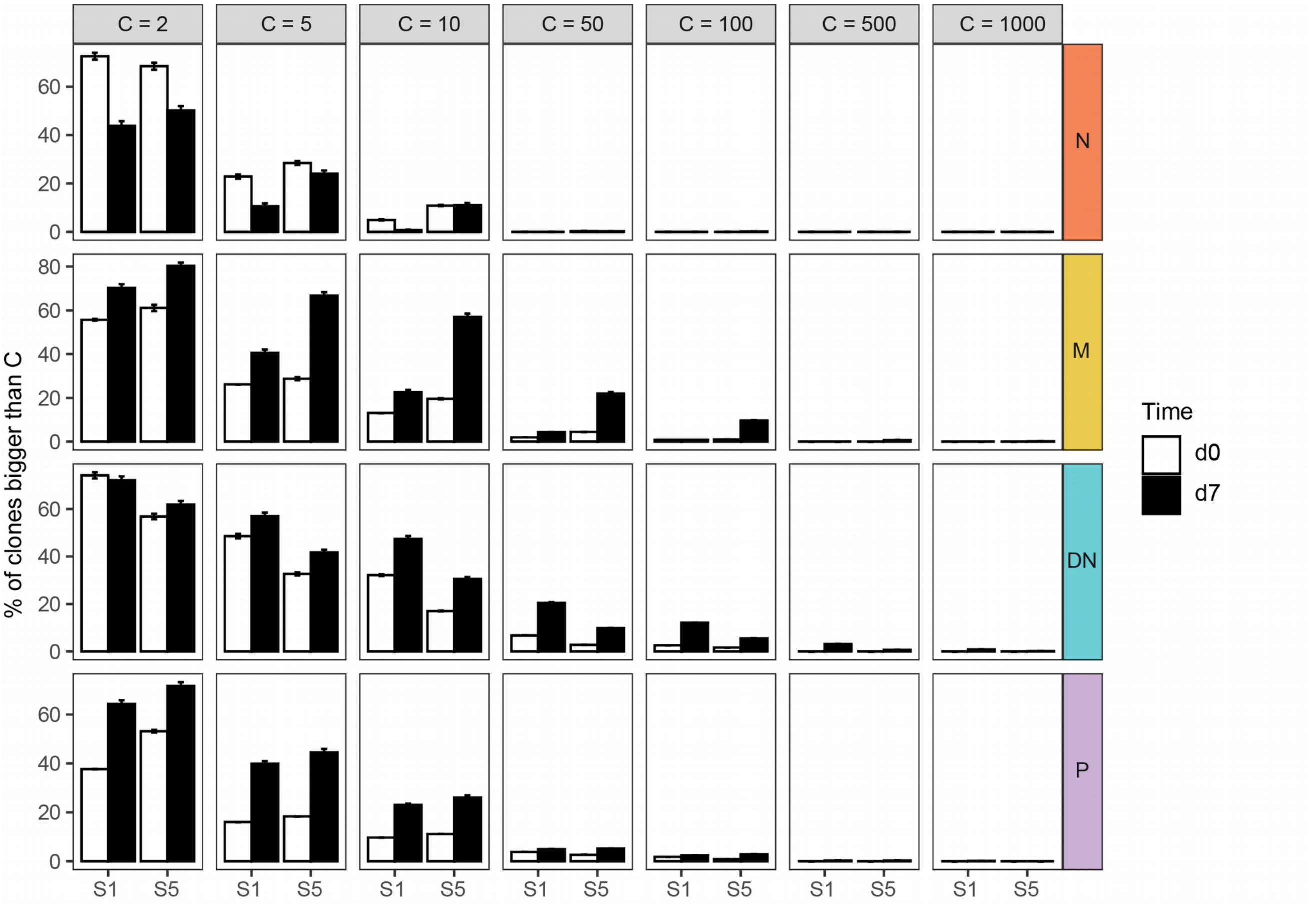

Fig. S5: Percentage of clones in the repertoire bigger or equal than size C, for different C values. To compute the percentages, a sampling of N=611 clones was performed according to their proportion in the repertoire. The error bars show the standard deviation of n=1000 bootstraps of the sampling.

Supplementary Figure S6

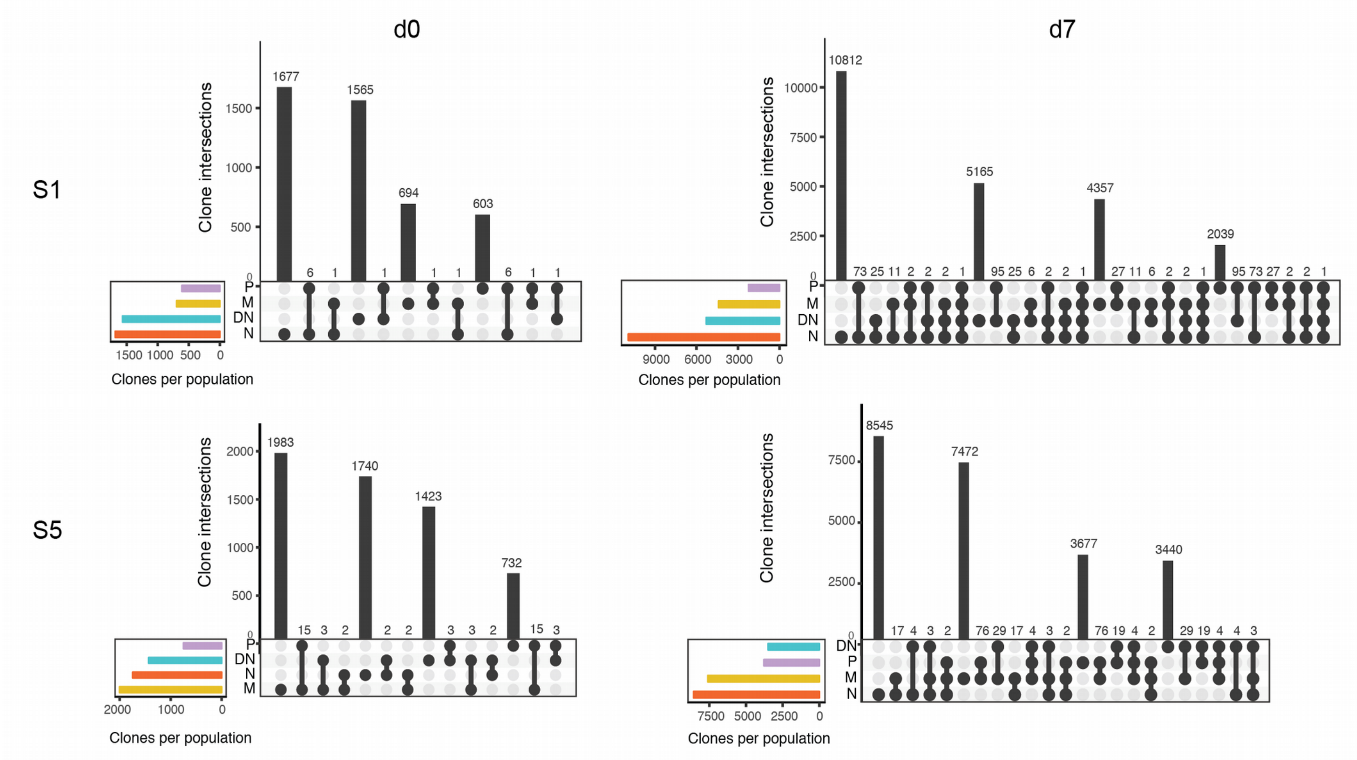

Fig S6: Upset plots representing the clonal overlaps among the B-cell populations of subjects 1 (S1) and 5 (S5) before and after vaccination (d7). Colored bars on the lower left for all the diagrams represent the total number of clones per population.

Supplementary Table S1: Detailed clinical characteristics of MS patients.

Patient	Disease subtype	Time since diagnosis (m)	Gender F / M	Age (y)	Time since start of relapse (d)	EDSS	Specific therapy at time-point of analysis
**MS 1**	CIS	0	M	27	3	2	none
**MS 2**	RMS	0	M	26	20	1	none
**MS 3**	RMS	62	F	27	3	2	none
**MS 4**	RMS	0	F	31	7	2	MPS
**MS 5**	CIS	0	F	30	7	0	none
**MS 6**	CIS	0	M	51	16	3	none
**MS 7**	CIS	0	F	43	14	4	MPS
**MS 8**	RMS	1	F	29	35	2	MPS
**MS 9**	CIS	0	M	27	7	0	MPS
**MS 10**	CIS	0	F	42	8	1	none
**MS 11**	RMS	66	F	29	6	2	MPS
**MS 12**	RMS	0	F	65	Stable	2.5	none
**MS 13**	RMS	3	F	22	90	2	none
**MS 14**	RMS	79	M	63	Stable	4	none
**MS 15**	RMS	54	M	41	Stable	1	none
**MS 16**	RMS	5	M	60	Stable	2.5	none
**MS 17**	RMS	30	M	40	Stable	2	none
**MS 18**	RMS	18	F	36	Stable	1	none
**MS 19**	RMS	0	M	60	6	2	none
**MS 20**	CIS	0	F	31	5	1	none
**MS 21**	CIS	0	M	37	stable	0	none

Abbreviations: CIS = clinically isolated syndrome, RMS = relapsing multiple sclerosis, F = female, M = male, MPS = methylprednisolone,

Supplementary Table S2. Genes of interest selected for the heatmap in Figure 2C. Adjusted p-values are indicated (p < 0.05, p < 0.01 or p < 0.001, ns = not significant) and the direction of regulation is indicated by colors (upregulated in green, down-regulated in red).

SYMBOL	Probe set ID	DN vs N	DN vs PB	DN vs M	PB vs N	M vs N	PB vs M
**SLAMF7**	16672654	ns	P < 0.01	ns	P < 0.001	ns	P < 0.001
**CR2**	16676795	ns	ns	ns	ns	ns	ns
**TRAF5**	16677133	ns	P < 0.001	ns	P < 0.001	ns	P < 0.001
**MTOR**	16681661	ns	ns	ns	ns	ns	ns
**IFNLR1**	16683551	ns	ns	ns	ns	ns	ns
**FCRL5**	16694886	ns	ns	ns	ns	ns	ns
**CXCR5**	16732037	ns	P < 0.001	ns	P < 0.001	ns	P < 0.001
**CD27**	16747261	0	P < 0.001	0	P < 0.001	P < 0.01	0
**FOXO1**	16778392	ns	ns	ns	ns	ns	ns
**GPR183**	16780592	ns	ns	ns	ns	ns	P < 0.001
**IGHD**	16797395	ns	ns	ns	P < 0.05	ns	ns
**IGHD**	16797425	ns	ns	ns	ns	ns	ns
**IGHD**	16797467	ns	ns	ns	ns	ns	ns
**IL4R**	16817254	ns	P < 0.001	ns	P < 0.001	P < 0.01	ns
**IL21R**	16817271	ns	ns	ns	ns	ns	ns
**CD19**	16817489	ns	ns	ns	P < 0.01	ns	P < 0.001
**ITGAX**	16818272	ns	ns	ns	ns	ns	ns
**IRF8**	16821621	ns	P < 0.01	ns	P < 0.001	ns	P < 0.001
**TBX21**	16835313	ns	ns	ns	ns	ns	ns
**CCR7**	16844381	ns	P < 0.001	ns	P < 0.001	ns	P < 0.001
**BCL2**	16855673	ns	ns	ns	ns	ns	ns
**ZEB2**	16903356	ns	ns	ns	ns	ns	ns
**IFIH1**	16904365	ns	ns	ns	ns	ns	ns
**IL10RB**	16904365	ns	ns	ns	ns	ns	ns
**BCL6**	16962584	ns	ns	ns	P < 0.001	ns	ns

**CD38**	16965268	ns	P < 0.05	ns	P < 0.01	ns	P < 0.001
**EBF1**	17002278	ns	P < 0.05	ns	P < 0.001	ns	P < 0.01
**IRF4**	17004167	ns	ns	ns	P < 0.001	ns	P < 0.001
**PRDM1**	17011279	ns	P < 0.01	ns	P < 0.001	ns	P < 0.001
**ABCB1**	17059491	ns	ns	ns	ns	ns	ns
**PAX5**	17093973	ns	P < 0.01	ns	P < 0.001	ns	P < 0.001
**TLR7**	17101531	ns	ns	ns	ns	ns	ns
**CD24**	17117110	ns	P < 0.001	ns	P < 0.001	ns	P < 0.001
**ZEB2**	17117888	ns	ns	ns	ns	ns	ns
**FCRL5**	17120182	ns	ns	ns	ns	ns	ns

Supplementary Table S3: Basic repertoire characteristics of targeted immunoglobulin repertoire analysis at baseline and day-7 after vaccination including isotype frequency, VH family usage and mutational analysis

		Vaccination subject 1							
		Baseline				Day 7			
		N	M	DN	P	N	M	DN	P
Isotype Frequency	*IgA %*	0	26	15	26	0	26	44	35
	IgD %	77	0	0	0	2	0	0	0
	IgG %	0	41	84	48	0	48	52	48
	IgM %	23	33	1	27	98	25	4	17
VH-Family Usage	*IGHV1 %*	18	19	10	7	29	17	15	5
	IGHV2 %	5	2	5	17	25	17	5	31
	IGHV3 %	23	28	31	22	16	28	49	19
	IGHV4 %	44	38	26	22	15	27	25	23
	IGHV5 %	8	13	26	32	14	11	6	23
Mutation Analysis	*Mean Mutation Count*	6	21	16	24	7	21	24	21
	SD Mutation Count	10	11	12	9	9	12	14	11

		Vaccination subject 5							
		Baseline				Day 7			
		N	M	DN	P	N	M	DN	P
Isotype Frequency	*IgA %*	0	27	46	24	0	18	30	27
	IgD %	42	0	0	0	12	0	0	0
	IgG %	0	67	44	48	0	81	62	57
	IgM %	58	6	10	28	88	1	8	16
VH-Family Usage	*IGHV1 %*	28	11	27	16	21	27	28	15
	IGHV2 %	9	37	8	20	28	25	10	22
	IGHV3 %	30	7	36	13	20	6	24	17
	IGHV4 %	20	14	16	18	15	23	29	23
	IGHV5 %	13	30	13	33	15	18	9	23
Mutation Analysis	*Mean Mutation Count*	9	16	14	19	9	21	17	20
	SD Mutation Count	13	11	12	11	14	12	12	11

Supplementary Table S4: Tick borne encephalitis ELISA test of recombinant antibodies (rAb) and serum

	**ELISA mean values**	**Interpretation**
**S1 day-0 (serum)**	38 VIEU/ml	Negative
**S1 day-14 (serum)**	352 VIEU/ml	Positive
**S5 day-0 (serum)**	111 VIEU/ml	Borderline
**S5 day-14 (serum)**	471 VIEU/ml	Positive
**S1 F4 day-9 (rAb)**	138 VIEU/ml	Positive
**S1 C3 day-9 (rAb)**	90 VIEU/ml	Borderline
**S5 D10 day-9 (rAb)**	Below detection limit	Negative

Negative < 63 VIEU/ml>; Borderline 63-126 VIEU/ml; Positive > 126 VIEU/ml

Supplementary Table S5 Cell counts, number of VH sequences and number of clones

		**Day-0**				**Day-7**			
		Cell counts	*Unique sequences with 2 representatives*	*Number of sequences after Igblast*	*Number of clones*	*Cell counts*	*Unique sequences with 2 representatives*	*Number of sequences after Igblast*	*Number of clones*
S1	N	65000	5818	4128	1684	350000	53409	21614	10928
	M	15000	5148	4441	696	310000	18931	16233	4406
	DN	9163	16317	11512	1566	48000	70578	53190	5296
	P	1009	6124	5221	611	1396	14417	12678	2239
S5	N	82000	8656	4887	1744	170000	34893	18594	8571
	M	61000	9528	8098	2003	110000	65015	55300	7603
	DN	25000	10800	7370	1431	84000	28797	23288	3499
	P	6251	4712	4209	750	10000	19654	17356	3778

S1 = subject 1; S5 = subject 5
